# Development and Validation of a Prognostic Model to Predict High-Risk Patients for Coronary Heart Disease in Snorers With Uncontrolled Hypertension

**DOI:** 10.3389/fcvm.2022.777946

**Published:** 2022-04-21

**Authors:** Meng-hui Wang, Mulalibieke Heizhati, Nan-fang Li, Xiao-guang Yao, Qin Luo, Meng-yue Lin, Jing Hong, Yue Ma, Run Wang, Le Sun, Ying-li Ren, Na Yue

**Affiliations:** ^1^Hypertension Center of People’s Hospital of Xinjiang Uygur Autonomous Region, Ürümqi, China; ^2^Xinjiang Hypertension Institute, Ürümqi, China; ^3^National Health Committee Key Laboratory of Hypertension Clinical Research, Ürümqi, China; ^4^Key Laboratory of Xinjiang Uygur Autonomous Region “Hypertension Research Laboratory”, Ürümqi, China; ^5^Xinjiang Clinical Medical Research Center for Hypertension (Cardio-Cerebrovascular) Diseases, Ürümqi, China

**Keywords:** coronary heart disease, hypertension, snorer, prognosis, nomogram

## Abstract

**Purpose:**

Snoring or obstructive sleep apnea, with or without uncontrolled hypertension, is common and significantly increases the risk of coronary heart disease (CHD). The aim of this study was to develop and validate a prognostic model to predict and identify high-risk patients for CHD among snorers with uncontrolled hypertension.

**Methods:**

Records from 1,822 snorers with uncontrolled hypertension were randomly divided into a training set (*n* = 1,275, 70%) and validation set (*n* = 547, 30%). Predictors for CHD were extracted to construct a nomogram model based on multivariate Cox regression analysis. We performed a single-split verification and 1,000 bootstraps resampling internal validation to assess the discrimination and consistency of the prediction model using area under the receiver operating characteristic curve (AUC) and calibration plots. Based on the linear predictors, a risk classifier for CHD could be set.

**Results:**

Age, waist circumference (WC), and high- and low-density lipoprotein cholesterol (HDL-C and LDL-C) were extracted as the predictors to generate this nomogram model. The C-index was 0.720 (95% confidence interval 0.663–0.777) in the derivation cohort and 0.703 (0.630–0.776) in the validation cohort. The AUC was 0.757 (0.626–0.887), 0.739 (0.647–0.831), and 0.732 (0.665–0.799) in the training set and 0.689 (0.542–0.837), 0.701 (0.606–0.796), and 0.712 (0.615–0.808) in the validation set at 3, 5, and 8 years, respectively. The calibration plots showed acceptable consistency between the probability of CHD-free survival and the observed CHD-free survival in the training and validation sets. A total of more than 134 points in the nomogram can be used in the identification of high-risk patients for CHD among snorers with uncontrolled hypertension.

**Conclusion:**

We developed a CHD risk prediction model in snorers with uncontrolled hypertension, which includes age, WC, HDL-C, and LDL-C, and can help clinicians with early and quick identification of patients with a high risk for CHD.

## Introduction

Coronary heart disease (CHD) is one of the main forms of cardiovascular disease and a leading cause of death and disease burden worldwide (1, 2). From a global perspective, the mean prevalence of CHD in adults older than 18 years is 6.03% [interquartile range (IQR) 3.70–7.60%], and this prevalence increases with age, reaching 19.34% (IQR 11.30–34.30%) among adults older than 65 years (3). Fortunately, CHD is a preventable non-communicable disease; therefore, strategic intervention for high-risk patients can effectively reduce the occurrence of CHD (4).

Snoring is a predictor of sleep apnea and a cardinal symptom of obstructive sleep apnea (OSA) (5), with a prevalence of approximately 7.9–56% in different populations and countries (6–8). Snoring is more common than OSA, accounting for 9–38% of population (9). Both of these conditions involve enhanced upper airway resistance during sleep (10), but OSA can only be distinguished from primary snoring with the evaluation by a physician and objective testing (11). Habitual snoring or the frequency of snoring is associated with the risk of hypertension in all age and sex groups (12), especially in midlife male snorers (13). Epidemiological and pathophysiological studies have revealed clear evidence of a causal relationship between OSA and hypertension (14–16) and also an increase in the hypertension risk with increasing severity of OSA (17). The involvement of snoring or OSA in poorly controlled hypertension may be owing to the activation of the sympathetic nervous system by upper respiratory tract resistance and intermittent hypoxia, subsequently inducing vasoconstriction, systemic vascular resistance, increased cardiac output, and elevated fluid retention, and finally leading to a sustained increase in blood pressure (BP) (12, 18, 19). Snoring or OSA, with or without uncontrolled hypertension, can increase cardiovascular morbidity and mortality (20–23).

An important strategy for primary prevention of CHD is the early identification of high-risk individuals (24). Useful methods for assessing patients’ risk of CHD include the use of prediction models, which can estimate a patient’s relative risk of outcomes and can help clinicians to decide whether to enhance management in certain individuals. Prediction models may be more effective than risk estimation based on BP level alone (25). Snorers have a 28% increased risk for coronary artery disease (20), and poor BP control has been a traditional predictor of CHD. Snoring and hypertension commonly coexist (15) and are associated with apnea, hypopnea, or hypoxemia, indicators that may be the predictors of CHD. However, population-based prediction models for CHD may not be appropriate or accurate when applied to snorers with uncontrolled hypertension owing to an absence of special parameters (26). Therefore, it is necessary to develop a prediction model to evaluate CHD risk in snorers with uncontrolled hypertension using characteristic predictors.

A variety of risk calculators are available to predict CHD, such as charts, Excel spreadsheets, algorithms, computer programs, and web-based tools. A nomogram is a novel way to present the results of an individualized prediction model because it is visual and can help clinicians better understand and apply prediction models. Therefore, the first aim of this study was to develop and internally validate a prognosis prediction model for CHD, which is displayed as a nomogram that is based on certain clinical features or routine biomedical or simple polysomnography (PSG) parameters. The second aim was to determine a risk classifier cutoff value of this model to identify patients with a high CHD, which can serve as a reference for clinicians in furthering education or strengthening patient management to prevent CHD.

## Materials and Methods

### Study Cohort

**FIGURE 1:**
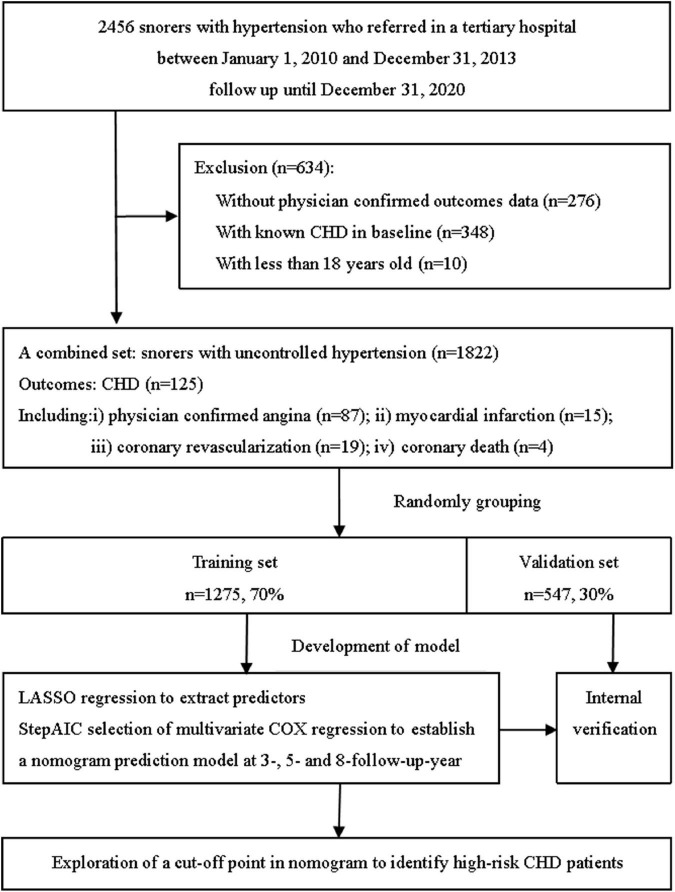
The flowchart of this study design.

Retrospective data with longitudinal follow-up information used in this study were obtained from the database of a single tertiary hospital. We reviewed the records of 2,456 inpatients with uncontrolled hypertension, who were admitted to the Hypertension Center of People’s Hospital of Xinjiang Uygur Autonomous Region referred from the community, primary care, or general clinic settings. All patients had undergone hypertension-related evaluation and PSG owing to self-reported or family members’ complaints of snoring at the Hypertension Center between January 1, 2010 and December 31, 2013. We excluded patients aged less than 18 years (*n* = 10), those with known CHD at baseline (*n* = 348), and those who were missing physician-confirmed outcome information (*n* = 276). Finally, we analyzed the data from 1,822 snorers with uncontrolled hypertension (Figure 1). Follow-up began at least 1 year after discharge and took a variety of forms, such as telephone contact or rehospitalization. The follow-up ended on December 31, 2020. The minimum follow-up was 1 year, and the longest was 10 years. Clinical data were collected and primary endpoints were identified and extracted from the medical records. The Institutional Ethics Committee of People’s Hospital of Xinjiang Uygur Autonomous Region approved the study design and data analysis and waived the need for informed consent owing to the retrospective nature of the study and use of anonymized data.

### Definitions

Uncontrolled hypertension was defined as systolic BP ≥ 140 mmHg and/or diastolic BP ≥ 90 mmHg in patients receiving antihypertensive treatment, regardless of the type of drugs. According to the medical records, CHD events were defined as physician-confirmed angina, myocardial infarction, coronary revascularization, and coronary death.

### Data Collection

The following clinical variables were collected from the electronic medical records on admission (Supplementary Table 1): ethnic, age, sex, smoking status, body mass index (BMI), neck circumference (NC), waist circumference (WC), hypertension duration, history of chronic respiratory diseases, diabetes mellitus (DM) presence, office BP on admission, and biochemical indicators that include fasting plasma glucose (FPG), serum total cholesterol (TC), high-density lipoprotein cholesterol (HDL-C), low-density lipoprotein cholesterol (LDL-C), triglycerides (TG), and high-sensitivity C-reactive protein (hs-CRP). We also calculated the estimated glomerular filtration rate (eGFR) as follows: 186 × (serum creatinine)^–1.154^ × (age in years)^–0.203^ × (0.742 if female sex). Biochemical indicators were measured *via* enzymatic methods using an auto-analyzer (7600-010 Automatic Analyzer: Hitachi Medical Systems, Suzhou, China). Some important factors influencing cardiovascular prognosis in hypertensive patients were also collected from the electronic medical records, including target organ damage (TOD) and concomitant clinical diseases (CCDs) (27). Specifically, TOD includes left ventricular hypertrophy, carotid ultrasound, or atherosclerotic plaque or stage 2–3 chronic kidney disease (CKD) (eGFR 30–59 mL/min/1.73 m^2^), and CCDs are defined as hemorrhage or ischemic stroke, lacunar infarction, atrial fibrillation, diabetic nephropathy, stage 4–5 CKD (eGFR < 30 mL/min/1.73 m^2^), retinal hemorrhage, or papillary edema. We collected sample PSG (Ultrasom, Nicolett, Madison, WI) parameters, including the apnea–hypopnea index (AHI) and lowest oxygen saturation (LSpO_2_). AHI was calculated using the number of obstructive apneas and hypopneas per hour of sleep. Apnea events were defined as the absence of airflow for >10 s, and hypopnea events were defined as any airflow reduction lasting for >10 s and resulting in arousal or oxygen desaturation ≥ 4%, as previously described (28).

### Development and Validation Sets

A total of 1,822 patients were randomly divided into two groups: the training set (*n* = 1,275, 70%) to construct the nomogram model and the validation set (*n* = 547, 30%).

### Statistical Analysis

Continuous variables are presented as mean ± standard deviation or median and interquartile range, and categorical variables are expressed as percentage. In the training set, clinical characteristics were compared between groups with and without CHD using Student’s *t*-test for normally distributed continuous variables, the Wilcoxon rank-sum test for non-normally distributed variables, and the chi-square test for categorical variables. Cox regression analyses were used to estimate the hazard ratio (HR), and the 95% confidence interval (CI), or *p*-value.

For missing data, we used multiple imputations based on the all indicators and CHD events. We created five imputed datasets for missing variables that were then combined across all datasets. Extreme values after interposition (e.g., a negative measured value) were shifted to the lowest line values of those indicators to prevent undue leverage effects.

Univariate Cox regression was used to quantify the association between candidate predictors and CHD in the training set. We used the least absolute shrinkage and selection operator (LASSO) regression model, which is a popular method for removing independent variables that are irrelevant or have multicollinearity to prevent overfitting when selecting the candidates. Before multivariate Cox proportional hazards (PH) regression analysis, variance inflation factors for each predictor were calculated to determine the collinearity, and the PH assumption was tested to verify that the HR did not change over time with *p* > 0.05. Backward stepwise selection (stepAIC) was used to obtain a simplified model in multivariate Cox regression analysis to help to determine the predictors from candidates. Based on this single-split verification, we performed another 1,000 bootstrap internal samples in the combined set, and the split ratio was still 7:3. Adding the original single-split verification, there were 1,001 splits in total. In the backward stepwise selection of multivariate Cox regression analyses, those variables more frequently included in the final model were predictors. According to a comparison of integrated discrimination improvement (IDI) and continuous net reclassification improvement (NRI) between before (model 1) and after (model 2) stepwise selection analysis in multivariable Cox regression, we determined a final prediction model to establish the nomogram. The number of events per variable (EPV) was also used to evaluate the sample size, according to CHD events per number of candidate predictors.

Model discrimination was quantified using Harrell’s c-statistic and area under the receiver 158 operating characteristic (ROC) curve (AUC) values, and 1,000 bootstrap resampling was used for unbiased evaluation of the nomogram. An AUC of 0.60–0.75 indicates possibly helpful discrimination, and an AUC greater than 0.75 indicates clearly useful discrimination (29). Calibration curves were plotted to describe the consistency between nomogram-predicted probability and actual CHD-free survival at 3, 5, and 8 years in the training and validation sets, respectively.

Once the nomogram model was successfully established, both the total points and linear predictor (lp) could be obtained, which is a relatively fixed part of the semiparametric model in the Cox regression formula:


$$S\,\left(t\right)=S\,0\,{\left(t\right)}^{\wedge }\,\text{exp}\,\left(\text{lp}\right)$$

In the above formula, S0(t) is the baseline survival function estimated from the data, and S(*t*) is the cumulative survival; then, cumulative incidence is equal to 1 − S(*t*). The total points corresponding to lp = 0 were set as the cutoff in risk stratification to determine the nomogram-predicted patients with a relatively low or high risk of developing CHD. After stratification, we compared the frequency of CHD occurrence between low-risk and high-risk groups and also calculated the 10-year cumulative incidence of CHD using different total risk scoring. Because Kaplan–Meier analysis may overestimate the outcome risk, competitive risk bias should be addressed, mainly considering non-CHD death as a competing event.

This article was prepared in accordance with the TRIPOD reporting checklist (30), and the methods were assessed with PROBAST (31). Tests were two-sided and 0.05 was set as the *p*-value for statistical significance. The statistical analyses were performed using IBM SPSS version 25.0 (IBM Corp., Armonk, NY, United States) or R version 4.1.0 (The R Project for Statistical Computing, Vienna, Austria).

## Results

### Clinical Characteristics of Snorers With Uncontrolled Hypertension

According to the available data, among 2,456 snorers with uncontrolled hypertension, 473 can be diagnosed with CHD (19.3%), with a mean follow-up of 7.0 (7.0–8.0) years. Of the 1,822 included patients, the median age at baseline was 46.7 years (range 18–83 years), data were analyzed, and no significant difference was found between the training and validation cohort for all characteristics and follow-up times (Table 1). In the group with 10-year follow-up, 125 cases of new-onset CHD occurred, accounting for 6.8%. In total, there are 23 candidate predictors; therefore, the EPV was equal to 5.43 (125/23). Our cohort had missing information on NC (*n* = 146, 8.0%), TC (*n* = 42, 2.3%), TG (*n* = 47, 2.6%), HDL-C (*n* = 46, 2.5%), LDL-C (*n* = 44, 2.4%), FPG (*n* = 64, 3.5%), and hs-CRP (*n* = 58, 3.2%); the interpolated data are shown in Supplementary Table 2.

**TABLE 1:** Comparison of characteristics between training and validation sets.

Variables	Combined set (*N* = 1822)		
	Training (*n* = 1,275)	Validation (*n* = 547)	*P* value
Ethnic Han (%)	816 (64.2)	350 (64.1)	0.968
Sex [male (%)]	869 (68.2)	352 (64.4)	0.113
Age (years)	46.5 ± 10.0	47.0 ± 9.9	0.327
BMI (kg/m^2^)	28.1 ± 3.8	28.1 ± 3.7	0.892
NC (cm)	40.0 ± 3.6	40.1 ± 3.5	0.523
WC (cm)	99.4 ± 10.4	99.3 ± 10.2	0.896
Current smoking [*n* (%)]	385 (30.2)	168 (30.7)	0.826
Hypertensive duration (years)	3.0 (1.0, 7.0)	3.0 (1.0, 7.0)	0.959
Single hypertension [*n* (%)]	444 (34.8)	186 (34.0)	0.736
Hypertension with TOD [*n* (%)]	436 (34.2)	176 (32.2)	0.403
Hypertension with CCD [*n* (%)]	395 (31.0)	185 (33.8)	0.233
DM presence [*n* (%)]	179 (14.0)	74 (13.5)	0.773
Chronic respiratory diseases [*n* (%)]	33 (2.6)	10 (1.8)	0.327
eGFR (ml/min/1.73 m^2^)	97.8 ± 21.1	98.3 ± 21.3	0.634
Office SBP in admission (mmHg)	146.2 ± 17.0	146.9 ± 16.3	0.397
Office DBP in admission (mmHg)	97.8 ± 11.5	97.4 ± 11.5	0.487
FPG (mmol/L)	5.2 ± 1.3	5.2 ± 1.6	0.311
Serum TC (mmol/L)	4.6 ± 1.2	4.5 ± 1.2	0.596
Serum TG (mmol/L)	2.3 (1.8, 2.9)	2.2 (1.8, 2.9)	0.447
Serum HDL-C (mmol/L)	1.1 ± 0.3	1.1 ± 0.3	0.795
Serum LDL-C (mmol/L)	2.6 ± 0.8	2.6 ± 0.8	0.215
Serum hs-CRP (mmol/L)	2.0 (0.9, 3.7)	1.9 (0.9, 3.7)	0.263
AHI (events/hour)	13.2 (5.2, 27.9)	13.0 (5.6, 26.8)	0.830
LSpO_2_ (%)	82.0 (77.0, 86.5)	82.0 (77.0, 86.0)	0.842
Coronary heart disease [*n* (%)]	83 (6.5)	42 (7.7)	0.825
Confirmed angina [*n* (%)]	56 (4.4)	31 (5.7)	
Myocardial infarction [*n* (%)]	3 (0.2)	1 (0.2)	
Coronary revascularization [*n* (%)]	11 (0.9)	4 (0.7)	
Coronary death [*n* (%)]	13 (1.0)	6 (1.1)	
Follow-up time (years)	7.0 (7.0, 8.0)	7.0 (7.0, 8.0)	0.873

*AHI, apnea hypopnea index; BMI, body mass index; CCD, concomitant clinical diseases; CHD, coronary heart disease; DBP, diastolic blood pressure; eGFR, glomerular filtration rate; FPG, fasting plasma glucose; HDL-C, high-density lipoprotein cholesterol; hsCRP, high-sensitivity C-reactive protein; LDL-C, low-density lipoprotein cholesterol; LSpO_2_, lowest oxygen saturation; NC, neck circumference; SBP, systolic blood pressure; TC, total cholesterol; TG, triglycerides; TOD, target organ damage; WC, waist circumference. Continuous variables are presented as mean ± standard deviation or medians and interquartile spacing and categorical variables are expressed as percentages. Student’s t-test (continuous variables) and Pearson’s chi-square test (categorical variables) were performed to compare between training and validation sets.*

### Predictors and Construction of the Nomogram Model

**FIGURE 2:**
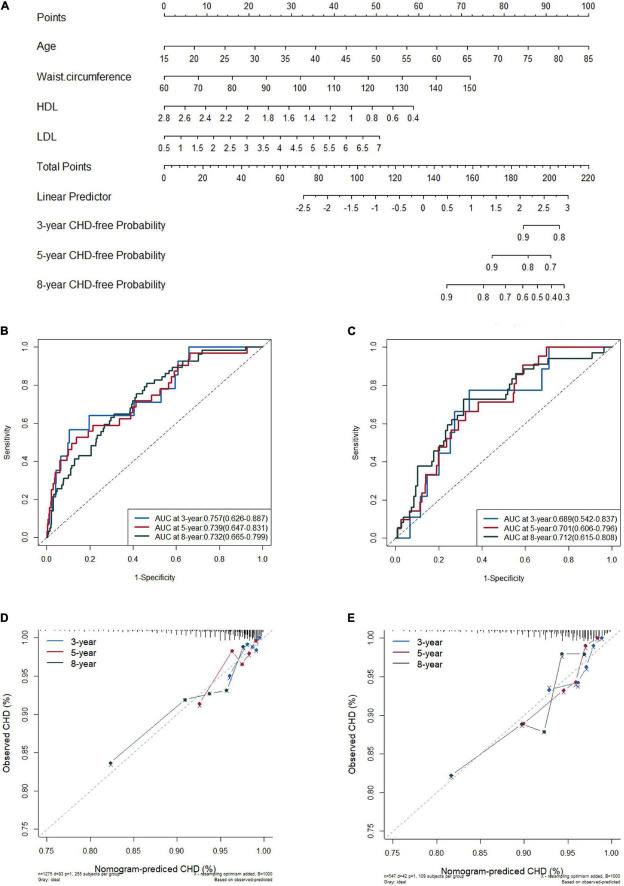
Nomogram for predicting new onset coronary heart disease (CHD) in the training set **(A)**, the receiver operating characteristic (ROC) curves of the model in training set **(B)** and in validation set **(C)**, and the calibration plots in the training set **(D)** and in the validation set **(E)** at 3, 5, and 8 years.

In the training set, patients with CHD were less ethic Han (*p* = 0.006), older (*p* < 0.001) and had higher values of BMI (*p* = 0.006), NC (*p* = 0.004), WC (*p* < 0.001), more hypertensive with TOD (*p* = 0.039) and DM (*p* = 0.038), AHI (*p* = 0.024), and LSpO2 (*p* = 0.027) than patients without CHD (Supplementary Table 3). We performed LASSO regression analysis and identified ten candidate predictors without multicollinearity in the training set: age, male, BMI, NC, WC, hypertension with TOD, DM, HDL-C, LDL-C, and AHI (Table 2, Supplementary Table 4, and Supplementary Figure 1). In the PH assumption test conducted prior to performing Cox regression analysis, the *p*-values of all predictors were >0.05 (Supplementary Table 5). After backward stepwise selection in multivariate Cox regression analysis, we obtained two models. Model 1 retained all predictors from the LASSO analysis; the AUC (95% CI) was 0.727 (0.670–0.783) at 8 years. In model 2, five predictors were extracted owing to the weaker association with CHD, including male, BMI, NC, DM and AHI, with AUC (95% CI) 0.720 (0.662–0.774) at 8 years. Comparisons of the AUC between the two models revealed no significant difference (Supplementary Figure 2). Continuous NRI and IDI revealed a slightly negative improvement from models 1 to 2 (-0.175 and -0.007, *p* > 0.05; Table 3). Model 1 maintains the characteristics of the specific group studied, such as larger NC and elevated AHI; however, as a practical predictive tool, model 2 is simpler and easier to use because it only involves five predictors, including age, WC, hypertension with TOD, HDL-C, and LDL-C (Table 3), as similar as that from the 1,000 bootstrap analyses. There were few differences in their coefficients between the two internal validation forms (Supplementary Table 6). Of these five predictors, CHD is weakly associated with hypertension with TOD [HR (95% CI): 1.51 (0.96–2.36), *p* = 0.073] in the COX regression analysis (Table 3). Then, we performed a further exploring analysis (Supplementary Figure 3). The results suggested that it is almost negligible about the median improvement of the model in risk score with or without hypertension with TOD (AUC: 0.720 vs. 0.717, *p* = 0.432). Therefore, the four predictors, including age, WC, HDL-C, and LDL-C, were selected to establish the final nomogram model (Figure 2A). The processes and methods of modeling, including ROC curve and optimal threshold analysis of prediction model, are shown in Supplementary Table 7.

**TABLE 2:** Univariable Cox regression analysis and least absolute shrinkage and selection operator (LASSO) regression analysis to extract the potential predictors in the training set.

Variables	Univariable cox regression		LASSO regression
	HR (95% CI)	*p-*value	Lambda (log) = 0.006 (−5.1193)
Age	1.05 (1.03, 1.07)	<0.0001	0.0473702184965897
Male	1.18 (0.73, 1.90)	0.4966	0.0000000000000001
BMI	1.07 (1.02, 1.13)	0.0047	0.0081677379934463
NC	1.08 (1.01, 1.15)	0.0185	0.0148402550539110
WC	1.04 (1.02, 1.06)	0.0002	0.0188237783613461
Current smoking	0.91 (0.57, 1.47)	0.7044	0
Hypertensive duration	1.04 (1.01, 1.07)	0.0136	0
Single hypertension	0.58 (0.35, 0.96)	0.0323	0
Hypertension with TOD	1.52 (0.98, 2.34)	0.0600	0.0055683324443603
Hypertension with CCD	1.08 (0.67, 1.73)	0.7502	0
DM presence	2.33 (1.38, 3.93)	0.0015	0.0027472424787100
Chronic respiratory diseases	1.53 (0.48, 4.85)	0.4701	0
eGFR	1.00 (0.99, 1.01)	0.5128	0
Office SBP	1.01 (0.99, 1.02)	0.3401	0
Office DBP	0.98 (0.96, 1.00)	0.0764	0
FPG	1.13 (0.98, 1.30)	0.1055	0
Serum TC	1.03 (0.86, 1.24)	0.7169	0
Serum TG	0.93 (0.77, 1.12)	0.4507	0
Serum HDL-C	0.49 (0.22, 1.10)	0.0856	**−**0.4454591060154290
Serum LDL-C	1.32 (1.01, 1.72)	0.0411	0.1889524482600690
Serum hsCRP	1.03 (0.97, 1.10)	0.3084	0
AHI	1.01 (1.00, 1.02)	0.0225	0.0007788607916679
LSpO_2_	0.99 (0.97, 1.00)	0.0542	0

*AHI, apnea hypopnea index; BMI, body mass index; CCD, concomitant clinical diseases; CHD, coronary heart disease; CI, confidence interval; DBP, diastolic blood pressure; eGFR, lomerular filtration rate; FPG, fasting plasma glucose; HDL-C, high-density lipoprotein cholesterol; HR, hazard ratio; hsCRP, high-sensitivity C-reactive protein; LASSO, least absolute shrinkage and selection operator; LDL-C, low-density lipoprotein cholesterol; LSpO_2_, lowest oxygen saturation; NC, neck circumference; SBP, systolic blood pressure; TC, total cholesterol; TG, triglycerides; TOD, target organ damage; WC, waist circumference. Tuning parameter (lambda) selection in the LASSO model used 10-fold crossvalidation.*

**TABLE 3:** Multivariate cox regression analysis to construct a nomogram model from randomly grouped data for prediction CHD in the training set.

Variables	Model 1			Model 2^#^		
	β	HR (95% CI)	*p*-value	β	HR (95% CI)	*p*-value
Age	0.063	1.06 (1.04, 1.09)	<0.0001	0.057	1.06 (1.04, 1.08)	<0.0001
Male	0.259	1.30 (0.67, 2.44)	0.423	–		
BMI	0.031	1.03 (0.93, 1.14)	0.541	–		
NC	0.016	1.01 (0.93, 1.11)	0.718	–		
WC	0.014	1.01 (0.98, 1.05)	0.467	0.032	1.03 (1.01, 1.05)	0.0013
Hypertension with TOD	0.347	1.41 (0.89, 2.25)	0.143	0.410	1.51 (0.96, 2.36)	0.0731
DM presence	0.227	1.25 (0.71, 2.22)	0.436	–		
Serum HDL-C	**−**0.796	0.45 (0.19, 1.10)	0.080	**−**0.949	0.39 (0.16, 0.93)	0.0330
Serum LDL-C	0.312	1.37 (1.04, 1.79)	0.023	0.291	1.34 (1.02, 1.75)	0.0323
AHI	0.003	1.00 (0.99, 1.01)	0.627	**–**		
*AUC (95% CI)	0.727 (0.670, 0.783)			0.720 (0.662, 0.778)		
^†^IDI (95% CI)				**−**0.007 (**−**0.049, 0.001), *p* = *0.066*		
^†^Continuous NRI (95% CI)				**−**0.175 (**−**0.300, 0.009), *p* = *0.060*		

AHI, apnea hypopnea index; AIC, Akaike information criterion; AUC, area under the curve for receiver operating characteristic curves; CHD, coronary heart disease; CI, confidence interval; HDL-C, high-density lipoprotein cholesterol; HR, hazard ratio; LDL-C, low-density lipoprotein cholesterol; IDI, integrated discrimination improvement; NC, neck circumference; NRI, net reclassification improvement; TOD, target organ damage; WC, waist circumference. ^#^Stepwise (stepAIC) selected model. *AUC at 8-year.

*^†^Model 1 as a reference.*

### Assessment of Nomogram Model Accuracy

The C-index was 0.720 (95% CI 0.663–0.777) in the derivation cohort and 0.703 (95% CI 0.630–0.776) in the validation cohort. In the training cohort, the AUC for the established nomogram was 0.757 (95% CI 0.626–0.887), 0.739 (95% CI 0.647–0.831), and 0.732 (95% CI 0.665–0.799) for 3-, 5-, and 8-year CHD-free survival (Figure 2B); these AUC values were 0.689 (95% CI 0.542–0.837), 0.701 (95% CI 0.606–0.796), and 0.712 (95% CI 0.615–0.808) in the validation cohort (Figure 2C), respectively. Time-dependent AUC and C-index of the nomogram model from 1,000 bootstraps were shown in Supplementary Figures 4, 5, respectively. With the extension of follow-up time from 3 to 8 years, the nomogram model’s AUC values of both training and validation sets were always above 0.68 (Supplementary Table 7). The calibration plots for 3-, 5-, and 8-year CHD-free survival showed acceptable consistency in the training (Figure 2D) and validation cohorts (Figure 2E) between the nomogram-predicted probability of CHD-free survival and the observed CHD-free survival.

### Risk Classifier for Coronary Heart Disease and Utility of Model

Based on the lp in the nomogram model, we calculated the total points for every patient and set a risk classifier according to a cutoff of lp = 0 in the training and validation sets.

The total points were calculated as follows (all formulas about nomogram model in Supplementary Table 8):


(1.429×age-21.429)+(0.800×WC-48.016)+(-24.468×HDL-C+ 68.511)+(7.800×LDL-C-3.900).

**FIGURE 3:**
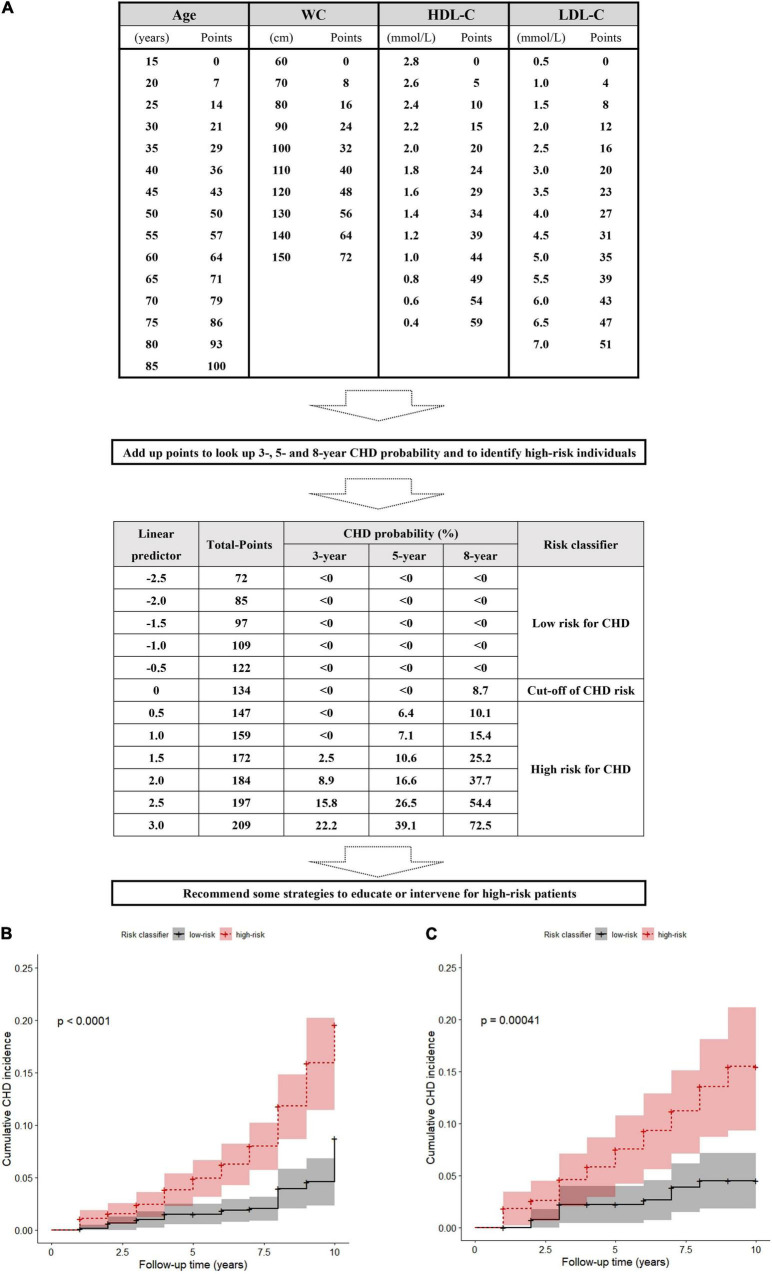
A score sheet and risk classifier to identify high-risk patients for CHD in snorers with uncontrolled hypertension **(A)**, and the cumulative incidences after risk stratification in the training set and **(B)** in the validation set **(C)**.

In the training set, if the lp was 0, the total was 134 points based on the nomogram. Patients with more than 134 total points were assigned to the group with a high risk of CHD. For further convenience of application, based on the above formula, the nomogram was converted to a score sheet for screening high-risk patients and evaluating total points and CHD probability (Figure 3A); the quantization table is provided in the Supplementary Excel algorithm. Regardless of which calculation method was used, patients with a total score of more than 134 points were predicted to be at high risk for CHD, thus requiring special attention and active management by clinicians.

### Cumulative Incidence

The actual CHD occurrence was significantly more frequent in the nomogram-predicted high-risk than the low-risk groups not only in the training set (9.76%, 62/635 vs. 3.28%, 21/640; χ^2^ = 20.009, *p* < 0.001; Supplementary Excel 1) but also in the validation set (10.84%, 31/286 vs. 4.21%, 11/261; χ^2^ = 8.448, *p* = 0.004; Supplementary Excel 2). Although we considered that competitive risk bias may influence estimates of CHD risk, after careful checking, only 2 of 1,822 patients died from non-CHD. Therefore, the result of competitive risk bias was insufficient to lead to the cumulative incidence misestimation of CHD events. The observed 10-year cumulative incidence of CHD in the nomogram-predicted high-risk group was also higher than that in the low-risk group in the training set (*p* < 0.0001; Figure 3B) and the validation set (*p* < 0.001; Figure 3C).

### Self-Assessment of Writing and Methodology Quality

Based on the PROBAST checklist, the overall risk assessment of the methodology was “high risk of bias” because of the retrospective cohort, small sample (EPV = 5.43), and the absence of mediating effect tests, crossvalidation, or external validation (Supplementary Table 9). According to the TRIPOD reporting checklist, writing quality self-assessment of this article is shown in Supplementary Table 10.

## Discussion

According to the published literature, the global prevalence of CHD ranges from 3.45 to 19.88% (3). In this study of snorers with uncontrolled hypertension, the total incidence rate of CHD was 19.3%, and the 10-year new-onset cumulative incidence was 6.86%. It should be noted that all patients with hypertension in this study had taken at least one antihypertensive drug, were hospitalized owing to concern for their health, and had good adherence to treatment. The incidence of CHD in patients with common snoring and hypertension may be much greater than the above rate. Therefore, this group is at high risk of CHD and requires greater attention from clinicians. Individualized management strategies should be developed according to the patients’ specific indicators. The prognosis model presented in this report involved only four commonly used indicators (age, WC, HDL-C, and LDL-C) to predict the risk of developing CHD within the subsequent decade among snorers with uncontrolled hypertension. This model offers a visual presentation that can help clinicians to better understand the results and apply the prediction model. The nomogram model had similar predictive accuracy in both training and validation cohorts, with AUCs of more than 0.7 and tolerable calibration. Using total points ≥ 134 in the nomogram, patients with a high risk of CHD can be identified and actively managed.

In the nomogram model, apart from WC, age, HDL-C, and LDL-C are known as traditional predictors for CHD (24). According to epidemiological data, the proportion of snorers with abdominal obesity is significantly higher (12) and the WC was positively correlated with the AHI (32). In a recent Mendelian randomization study, the results revealed that WC was causally associated with CHD, and a 12.5-cm increase in WC predicted more than a 1.5-fold increased risk of CHD (33). Therefore, WC is a powerful predictor of CHD that is reflected in the physical characteristics of snorers and can easily be measured (34). NC is also a user-friendly indicator of a relationship with CHD (35); unfortunately, NC was eliminated in the model after performing backward stepwise selection. Although we included AHI in our analysis, which can represent snoring or OSA severity (11), the AHI may contribute less to CHD than traditional predictors of CHD. In addition, the results based on the Cox regression analysis revealed that hypertension with TOD is more weakly associated with CHD than the other four predictors. Moreover, given that information on hypertension in association with organ damage is not readily available without a comprehensive assessment of the patient, the model’s generalizability and applicability will be limited. To prioritize simplicity of the risk model, only four easily obtainable indexes were retained in the final nomogram model. According to NRI and IDI assessments, the results showed that the accuracy of this simplified model in predicting CHD did not decrease significantly.

Although the nomogram is intuitive, its calculation requires measuring the points of each predictor, which is time-consuming in the clinic. Therefore, based on the known formula, the nomogram model can be translated into a score sheet and an Excel spreadsheet for clinicians to quickly obtain a rough determination of whether a patient is at high risk for CHD. Formulae to calculate the total points of the nomogram and probability of CHD occurrence are provided in Supplementary Material, which can help researchers evaluate patient data in batches or validate our model.

A difficulty in this study was determining cutoff points to distinguish high-risk patients for CHD. If ROC curves were used to identify these cutoff points, we could obtain multiple different cutoff values for different follow-up years. The non-uniformity among cutoff values makes this model difficult to use and affects the users’ understanding. Therefore, we used the semiparametric characteristic of the Cox regression model to solve this issue because the estimation of the lp value is a relatively stable part of this equation. Once the Cox model is established, the partial regression coefficients for each predictor can be determined, and the patient’s lp can be calculated according to the sum of weighted observations for each predictor, which corresponds to the total points in the nomogram. The total points estimated from baseline data at follow-up initiation will not change over time. Additionally, according to the principle that combined predictors increase hazards when lp is greater than 0, we suggest that the total points (*n* = 134) corresponding to lp = 0 are the cutoff values for identifying high-risk patients. This is consistent with what we predicted, that is, that the incidence of actual CHD was significantly increased among high-risk patients with lp > 0 in comparison with low-risk ones.

The strength of this study is that, for snorers with uncontrolled hypertension, patients with a high risk for CHD can be identified early and quickly according to the individuals’ age, WC, HDL-C, and LDL-C values. We maintained the continuous nature of each predictor to minimize the loss of patient’s information. To illustrate how this model can be applied, we can use a clinical example. A 40-year-old (36 points) man has failed to lower his BP to below 149/90 mmHg after regularly taking two antihypertensive drugs; he has self-reported snoring, WC 110 cm (40 points), HDL-C 0.8 mmol/L (49 points), and LDL-C 3.3 mmol/L (22 points). According to our nomogram, score sheet, or Excel algorithm, his total points are 147 (*n* > 134); this patient would therefore be identified as a patient with a high risk for CHD. Clinicians can then target this patient’s abnormal indicators, such as central obesity and hyperlipidemia, with reasonable use of antihypertensive drugs, informing the patient of the high risk of CHD and providing suggestions and a diet and exercise prescription and also lipid-lowering therapy. Establishment of health records and a follow-up schedule are needed to properly manage this patient. Adherence should be evaluated, and the patient should be reminded to see a doctor and undergo cardiac examinations to prevent irreversible ischemic heart disease if the patient experiences any chest discomfort.

This study includes several limitations. (i) According to PROBAST, the overall risk assessment of the methods used showed a “high risk of bias” owing to the use of a retrospective cohort, small sample (EPV < 10) and the absence of external validation. (ii) We must acknowledge that the nomogram’s ability to correctly distinguish CHD is limited because the AUC was less than 0.75. (iii) This was a single-center study with data from a tertiary hospital. Although many patients were referred from primary hospitals for uncontrolled hypertension, extensive use of the model in the general patient population is limited. (iv) Selection bias is inevitable in retrospective studies, especially when excluding some patients without baseline or follow-up outcome data. (v) Xinjiang province is a multiethnic region. Although we had complete data on all patients’ ethnicity, it was not included in the candidate predictors, considering from a non-population cohort and the hospitalization bias. (vi) OSA mainly increases nighttime systolic and 24-h diastolic BP variability in patients with hypertension, which may be a major risk determinant of cardiovascular diseases (36). Although ambulatory-monitored blood pressure (ABPM) data may provide a more precise prediction model for OSA patients, the ABPM is not yet an easily accessible measurement, involving which may limit the scope of this nomogram model application. Whereas office BP at admission is more random without being adjusted by hypertensive specialists, and thus results can be generalized more. And last but not least, DM presence, as a recognized predictor of CHD, was not included in the final nomogram model. The possible reason is that some patients have not developed diabetes at baseline but may gradually become diabetic during the 10-year follow-up. Therefore, the use of baseline data analysis may underestimate the impact of diabetes on CHD outcomes. In addition, besides DM and FPG, we also considered postprandial blood glucose or glycosylated hemoglobin as representative indicators of hyperglycemia, whereas there were a lot of missing data on these parameters, which unfortunately limited our analysis.

In summary, we developed a risk prediction model of CHD including age, WC, HDL-C, and LDL-C, for snorers with uncontrolled hypertension, which may help clinicians to identify patients with a high risk of CHD. Given that snoring and uncontrolled hypertension are both common, this risk-scoring system is useful for application in clinical practice in primary healthcare clinics. The accuracy and applicability of the model should be verified in further cohort studies.

## Data Availability Statement

The original contributions presented in the study are included in the article/Supplementary Material, further inquiries can be directed to the corresponding author/s.

## Author Contributions

N-FL and M-HW conceptualized the study. M-HW, MH, and X-GY contributed to methodology. JH, YM, RW, LS, Y-LR, and NY contributed to the resources and data curation. M-HW and MH contributed to writing – original draft preparation. QL and M-YL contributed to software. N-FL contributed to project administration and funding acquisition. All authors have read and agreed to the final version of the manuscript.

## Conflict of Interest

The authors declare that the research was conducted in the absence of any commercial or financial relationships that could be construed as a potential conflict of interest.

## Publisher’s Note

All claims expressed in this article are solely those of the authors and do not necessarily represent those of their affiliated organizations, or those of the publisher, the editors and the reviewers. Any product that may be evaluated in this article, or claim that may be made by its manufacturer, is not guaranteed or endorsed by the publisher.
