# Supplementary material for: Development and Validation of a Prognostic Model to Predict High-Risk Patients for Coronary Heart Disease in Snorers With Uncontrolled Hypertension
Source: Front Cardiovasc Med. 2022 Apr 21;9:777946. doi: 10.3389/fcvm.2022.777946 (PMC9069207; doi:10.3389/fcvm.2022.777946)
Supplement: Supplementary file 1 [file Data_Sheet_1.pdf]

Supplementary Material

Title: Development and Validation of a Prognostic Model to Predict High-risk Patients for Coronary Heart Disease in Snorers with Uncontrolled Hypertension

Authors: Meng-hui Wang, Mulalibieke Heizhati, Nan-fang Li, Xiao-guang Yao, Qin luo, Meng-yue Lin, Jing Hong, Yue Ma, Run Wang, Le sun, Ying-li Ren, Na Yue

Supplemental material: Some additional information about this work

Supplementary Table 1 Details of the clinical indicators

Variable	Abbreviation	Measurement/definition	Reference
Ethnic Han	-	Admission record	
Age [years]	-	Admission record	
Sex	-	Admission record	
Body mass index [kg/m^2]	BMI	Weight per (height)2	
Neck circumference [cm]	NC	At the narrowest part of the neck, the length around the neck.	Male <39 female<35
Waist circumference [cm]	WC	At the position of two fingers on the belly button, the length around the waist	Male <85 female<80
Current smoking	-	At admittion without quit smoking	
Hypertensive duration [years]	-	Patient self-reported time from discovery of hypertension to admission	
Hypertensive severity			
Single hypertension	-	Discharge records did not indicate any organ damage	
With target organ damage	With TOD	Including left ventricular hypertrophy, carotid ultrasound, or atherosclerotic plaque, or chronic kidney disease 2-3 stage (eGFR: 30-59 ml/min/1.73 m2)	
With concomitant clinical diseases	With CCD	Including hemorrhage or ischemic stroke, lacunar infarction, atrial fibrillation, diabetes mellitus, diabetic nephropathy, CKD 4-5 stage (eGFR < 30 ml/min/1.73 m2), retinal hemorrhage or papillary edema	
Chronic respiratory diseases	CRD	A record of past history	
Estimated-glomerular filtration rate [ml/min/1.73 m2]	eGFR	186 × (serum creatinine) −1.154 × (age) −0.203 × (0.742 if female)	about 125
Office systolic blood pressure [mmHg]	Office SBP	Records from the first day of admission	< 140
Office diastolic blood pressure [mmHg]	Office DBP	Records from the first day of admission	< 90
Fasting plasma glucose [mmol/L]	FPG	Biochemical indicators were fasting blood-collection indexes, measured by enzymatic methods using an auto-analyzer (7600-010 Automatic Analyzer: Hitachi Medical Systems, Suzhou, China)	3.9-6.1
Total cholesterol [mmol/L]	TC		2.59-6.47
Triglycerides [mmol/L]	TG		0-1.7
High density lipoprotein cholesterol[mmol/L]	HDL-C		>1.04
Low density lipoprotein cholesterol [mmol/L]	LDL-C		0-3.37
High sensitivity C-reactive protein [mmol/L]	hs-CRP		0-5
Apnea hypopnea index [events/hour]	AHI	Polysomnography (Ultrasom, Nicolett, Madison, WI)	<5
Lowest oxygen saturation [%]	$LSpO_2$		≥ 90

Supplementary Table 2 Interpolation of missing data

Variables of missing data	Before interpolation		After interpolation		P# value
	n	Mean(SD)/Median (Q1-Q3)	n	Mean(SD)/Median (Q1-Q3)	
NC [cm]	1676	39.894 ± 3.821	1822	39.901 ± 3.783	0.984
FPG [mmol/L]	1758	5.221 ± 1.424	1822	5.225 ± 1.390	0.908
TC [mmol/L]	1780	4.527 ± 1.181	1822	4.528 ± 1.170	0.965
TG [mmol/L]	1775	1.690 (1.210-2.390)	1822	1.700(1.210-2.390)	0.783
HDL-C[mmol/L]	1776	1.119 ± 0.298	1822	1.119 ± 0.296	0.995
LDL-C [mmol/L]	1778	2.609 ± 0.796	1822	2.610 ± 0.789	0.946
hsCRP [mmol/L]	1764	1.950 (0.860-3.770)	1822	1.980 (0.880-3.820)	0.586

Pairwise test was used to compare the differences before and after interpolation

Supplementary Table 3 Comparison of characteristics between with and without CHD groups in the training or validation set

**Variables**	**Training set**				**Validation set**		
	**With CHD (n=83)**	**Without CHD (n=1192)**	***P* value**		**With CHD (n=42)**	**Without CHD (n=505)**	**P value**
Ethnic Han [n (%)]	41 (50.0%)	775 (65.2%)	0.006		57 (46.0%)	1109 (65.5)	<0.001
Age [years]	51.5 ± 10.9	46.2 ± 9.9	<0.001		51.6 ± 11.0	46.6 ± 9.8	0.002
Sex [male (%)]	59 (71.1%)	810 (68.0%)	0.554		28 (66.7%)	324 (64.2%)	0.744
BMI [kg/m2]	29.2 ± 4.1	28.0 ± 3.8	0.006		29.0 ± 4.1	28.0 ± 3.7	0.079
Neck circumference [cm]	40.9 ± 3.4	39.9 ± 3.6	0.023		41.0 ± 4.1	40.0 ± 3.5	0.091
Waist circumference [cm]	103.3 ± 11.5	99.1 ± 10.3	<0.001		102.5 ± 10.3	99.1 ± 10.2	0.039
Current smoking [n (%)]	24 (28.9%)	361 (30.3%)	0.793		15 (35.7%)	153 (30.3%)	0.465
Hypertensive duration [years]	4.0 (2.0-9.0)	3.0 (1.0-7.0)	0.058		4.5 (0.6-10.0)	3.0 (1.0-7.0)	0.329
Single hypertension [n (%)]	21 (25.3%)	423 (35.5%)	0.060		13 (31.0%)	173 (34.3%)	0.072
Hypertensive with TOD [n (%)]	28 (33.7%)	311 (26.1%)	0.039		9 (21.4%)	127 (25.1%)	0.031
Hypertensive with CCD [n (%)]	34 (41.0%)	458 (38.4%)	0.861		20 (47.6%)	205 (40.6%)	0.722
DM presence [n (%)]	18 (21.7%)	161 (13.5%)	0.038		28 (22.4%)	225 (13.3%)	0.004
Chronic respiratory diseases [n (%)]	3 (3.6%)	30 (2.5%)	0.543		2 (4.8%)	8 (1.6%)	0.140
eGFR [ml/min/1.73 m2]	96.3 ± 22.3	97.9 ± 21.0	0.492		97.1 ± 18.6	98.4 ± 21.5	0.693
Office SBP [mmHg]	147.8 ± 18.1	146.1 ± 17.0	0.382		150.2 ± 15.5	146.7 ± 16.3	0.174
Office DBP [mmHg]	95.9 ± 11.2	98.0 ± 11.5	0.111		94.4 ± 12.1	97.7 ± 11.4	0.072
Serum glucose [mmol/L]	5.3 ± 1.4	5.2 ± 1.3	0.401		5.7 ± 1.9	5.2 ± 1.6	0.040
Serum cholesterol [mmol/L]	4.6 ± 1.1	4.6 ± 1.2	0.763		4.8 ± 1.8	4.5 ± 1.1	0.079
Serum triglycerides [mmol/L]	2.3 (1.8-2.9)	2.3 (1.8-2.8)	0.981		2.3 (1.8-3.2)	2.2 (1.8-2.9)	0.505
Serum HDL [mmol/L]	1.1 ± 0.3	1.1 ± 0.3	0.162		1.1 ± 0.2	1.1 ± 0.3	0.144
Serum LDL [mmol/L]	2.8 ± 0.8	2.6 ± 0.8	0.053		2.7 ± 1.1	2.6 ± 0.8	0.523
Serum hsCRP [mmol/L]	1.7 (0.7-4.5)	2.0 (0.9-3.6)	0.520		2.5 (0.9-4.9)	1.9 (0.9-3.7)	0.360
AHI [events/hour]	15.9 (7.9-31.4)	13.1 (4.8-27.5)	0.024		14.0 (5.8-26.6)	13.0 (5.6-26.7)	0.806
Lowest oxygen saturation [%]	81.0 (73.0-85.0)	82.0 (77.0-87.0)	0.027		82.0 (76.0-86.5)	82.0 (77.0-86.0)	0.596

Continuous variables are presented as mean ± standard deviation or medians and inter-quartile spacing and categorical variables are expressed as percentages. Student' t test (continuous variables) and Pearson' chi-square test (categorical variables) was performed to compare between with and without CHD in training or validation sets

AHI apnea hypopnea index, *B*MI body mass index, CCD concomitant clinical diseases, *CHD* coronary heart disease, DBP diastolic blood pressure, eGFR glomerular filtration rate, *HDL-C* high density lipoprotein cholesterol, *hsCRP* high sensitivity C-reactive protein, *LDL-C* low density lipoprotein cholesterol, *SBP* systolic blood pressure, *TOD* target organ damage

Supplementary Table 4 Col- linearity analysis of predictors

Predictors	Variance inflation factor
Age	1.2
Male	1.8
BMI	3.0
Neck circumference	2.3
Waist circumference	3.3
Hypertensive with TOD	1.1
DM presence	1.1
Serum high density lipoprotein cholesterol	1.2
Serum low density lipoprotein cholesterol	1.0
Apnea hypopnea index	1.2

Supplementary Table 5 Proportional hazards (PH) assumption test of predictors before multivariate Cox regression analysis

Predictors	chi-square	P value
Age	0.7558	0.38
Male	1.2303	0.27
BMI	0.1264	0.72
Neck circumference	1.3339	0.25
Waist circumference	2.5840	0.11
Hypertensive with TOD	1.8826	0.17
DM presence	0.8030	0.37
Serum high density lipoprotein cholesterol	1.2169	0.27
Serum low density lipoprotein cholesterol	2.2990	0.13
Apnea hypopnea index	0.0705	0.79
Global	10.8716	0.37

Supplementary Table 6 Predictors selection from a single split and 1000 bootstrap resampling validation approach based on multivariate Cox regression analysis

Variables	**β coefficient (mean)**			**HR (mean (95%CI))**	
	A single split	1000 bootstrap		A single split	1000 bootstrap
Age	0.0626	0.0638		1.065(1.040~1.090)	1.066(1.038~1.097)
Male	0.2587	0.3034		1.295(0.688~2.439)	1.355(0.732~2.529)
BMI	0.0309	0.0331		1.031(0.934~1.139)	1.034(0.944~1.137)
NC	0.0160	0.0156		1.016(0.931~1.109)	1.016(0.938~1.096)
WC	0.0139	0.0138		1.014(0.977~1.053)	1.014(0.978~1.050)
Hypertension with TOD	0.3471	0.3468		1.415(0.889~2.251)	1.416(0.860~2.260)
DM presence	0.2267	0.2239		1.255(0.709~2.220)	1.251(0.610~2.236)
HDL-C	-0.7955	-0.8557		0.451(0.185~1.101)	0.425(0.144~1.187)
LDL-C	0.3123	0.3228		1.367(1.044~1.789)	1.381(1.061~1.789)
AHI	0.0026	0.0020		1.003(0.992~1.013)	1.002(0.860~1.014)
**Backward stepwise selection of multivariate Cox regression analyses**					
Age	0.0602	0.0640		1.062(1.040~1.085)	1.066(1.046~1.092)
WC	0.0296	0.0309		1.030(1.001~1.051)	1.031(1.017~1.045)
Hypertension with TOD	0.4097	0.5691		1.506(0.962~2.357)	1.767(1.417~2.521)
HDL-C	-0.9490	-1.0949		0.387(0.162~0.926)	0.335(0.119~0.541)
LDL-C	0.2909	0.3485		1.338(1.025~1.746)	1.417(1.215~1.620)

AHI = apnea hypopnea index, CCD = concomitant clinical diseases, CI = confidence interval, HDL-C = high density lipoprotein cholesterol, LDL-C = low density lipoprotein cholesterol, NC = neck circumference, HR = hazard ratio, TOD = target organ damage, WC = waist circumference

Supplementary Table 7 ROC curve analysis of prediction model and optimal threshold analysis

Time (years)	Best.cut.X	Best.cut. sensitivity	Best.cut. specificity	CHD (n)	Censored (n)	Cumulative Incidence of CHD	Predicted CHD-free probability	AUC
Full model from 1000 bootstrap in the training set								
3	5.853365	0.710	0.681	14	131	0.011	0.9829	0.760
4	5.853365	0.712	0.685	21	164	0.017	0.9733	0.757
5	5.853365	0.685	0.687	32	188	0.027	0.9680	0.746
6	5.853365	0.710	0.691	38	226	0.032	0.9597	0.762
7	5.998062	0.745	0.631	47	654	0.040	0.9502	0.742
8	5.853365	0.663	0.688	57	939	0.050	0.9221	0.734
Stepwise most selected model from 1000 bootstrap in the training set								
3	5.9256689	0.927	0.536	14	131	0.011	0.9829	0.772
4	5.1084255	0.569	0.851	21	164	0.017	0.9733	0.763
5	5.1084255	0.528	0.851	32	188	0.027	0.9680	0.741
6	5.6315745	0.737	0.668	38	226	0.032	0.9597	0.756
7	5.8647674	0.807	0.557	47	654	0.040	0.9502	0.731
8	5.6315745	0.681	0.659	57	939	0.050	0.9221	0.722
Full model from 1000 bootstrap in the validation set								
3	6.2155495	0.887	0.514	9	52	0.017	0.9659	0.710
4	6.2270882	0.945	0.520	18	58	0.034	0.9599	0.730
5	6.0417402	0.858	0.632	21	72	0.040	0.9518	0.741
6	6.0417402	0.839	0.642	25	89	0.048	0.9413	0.750
7	6.2270882	0.897	0.556	30	258	0.059	0.9261	0.751
8	6.0529265	0.750	0.676	37	402	0.074	0.9118	0.717
Stepwise most selected model from 1000 bootstrap in the validation set								
3	5.9298973	0.887	0.514	9	52	0.017	0.9659	0.678
4	5.9298973	0.888	0.524	18	58	0.034	0.9599	0.707
5	5.9298973	0.905	0.533	21	72	0.040	0.9518	0.721
6	5.9298973	0.921	0.540	25	89	0.048	0.9413	0.742
7	5.9843364	0.899	0.548	30	258	0.059	0.9261	0.740
8	5.9843364	0.832	0.583	37	402	0.074	0.9118	0.720

Supplementary Table 8 Formulas to calculate points of every predictors and CHD probability at 3-, 5- and 8- follow-up-year

Predictors	Formulas to calculate points of every predictors	Total points
Age (years)	Points①:1.428571429*Age-21.428571429	①+②+③+④
WC (cm)	Points②:0.800267076*WC-48.016024573	
HDL-C (mmol/L)	Points③:-24.468209409*HDL+68.510986345	
LDL-C (mmol/L)	Points④:7.80028582*LDL-3.90014291	
CHD probability	Formulas to calculate CHD probability at 3-, 5- and 8- year	
at 3-year	-0.005342045*Total points+1.894131923	
at 5-year	-2.44e-07*Total points ^3+3.1227e-05*Total points^2+0.007047799*Total points	
at 8-year	3.21e-07*Total points ^3-0.000277943*Total points^2+0.058050376*Total points-2.647611113	

CHD coronary heart disease, *HDL-C* high density lipoprotein cholesterol, *LDL-C* low density lipoprotein cholesterol, *WC* waist circumference

Supplementary Table 9 Self-assessment of methodological quality based on the PROAST

Question	Assessment	Explanation
1: Participants		
1·1 Were appropriate data sources used, e.g., cohort, RCT or nested case-control study data?	Probably no	It's a prognostic prediction model studies, we used a retrospective data with longitudinal follow-up information to analysis.
1·2 Were all inclusions and exclusions of participants appropriate?	Yes	The inclusion and exclusion criteria were reasonable and consistent with those described in methods.
Overall risk of bias of issue 1	High risk of bias	
2: Predictors		
2·1 Were predictors defined and assessed in a similar way for all participants?	Yes	This study was a single-center study, and predictors were based on patient physiological characteristics and laboratory tests, with uniform definitions.
2·2 Were predictor assessments made without knowledge of outcome data?	Yes	All patients known CHD outcome were excluded before analysis,therefore, observed CHD events were new onset.
2·3 Are all predictors available at the time the model is intended to be used?	Yes	All indicators were analyzed in LASSO regression, and all predictors was selected to establish the nomogram model.
Overall risk of bias of issue 2	Low risk of bias	
3: Outcome		
3·1 Was the outcome determined appropriately?	Yes	Diagnostic outcomes were coronary heart disease(CHD) (Yes/No).
3·2 Was a pre-specified or standard outcome definition used?	Yes	Diagnostic outcomes were consistent with guidelines or journal approved classification methods.
3·3 Were predictors excluded from the outcome definition?	Yes	Predictors are not included in the CHD diagnostic criteria.
3·4 Was the outcome defined and determined in a similar way for all participants?	Yes	The definition of PA was the same for all subjects.
3·5 Was the outcome determined without knowledge of predictor information?	Yes	Predictors were baseline data, and information on outcome was available only after several years of longitudinal follow-up.
3·6 Was the time interval between predictor assessment and outcome determination appropriate?	Yes	All follow-up began one year after discharge.
Overall risk of bias of issue 3	Low risk of bias	
4: Analysis		
4·1 Were there a reasonable number of participants with the outcome?	No	The total number of candidate variables was 7, and the number of PA patients was 125, so the number of events per variable (EPV) =125/20=6.25, which was a small sample.
4·2 Were continuous and categorical predictors handled appropriately?	Yes	Except for gender, which was the natural 2 classification variable, other continuous variables were not converted into classification variables.
4·3 Were all enrolled participants included in the analysis?	Yes	All patients who met the inclusion and exclusion criteria were included in the analysis.
4·4 Were participants with missing data handled appropriately?	Yes	For missing data, we used multiple imputation on the basis of all indicators and CHD events.
4·5 Was selection of predictors based on univariable analysis avoided?	Yes	The candidates selection was based on LASSO regression analysis.
4·6 Were complexities in the data (e.g., censoring, competing risks, sampling of controls) accounted for appropriately?	Yes	Information on whether complexity exists or is properly accounted for in the data is not reported.
4·7 Were relevant model performance measures evaluated appropriately?	Yes	Discrimination was assessed by AUC, and calibration curve was used to assess calibration.
4·8 Were model overfitting and optimism in model performance accounted for?	Probably no	Mediating effect tests or cross-validation are not included
4·9 Do predictors and their assigned weights in the final model correspond to the results from multivariable analysis?	Yes	The weights assigned to the predictors in the nomogram model are consistent with the results of multivariate analysis.
Overall risk of bias of issue 4	High risk of bias	
Overall risk of bias of issue 1-4	High risk of bias	Retrospective cohort, small sample and the absence of mediating effect tests, cross-validation or external validation existed in paper.

Supplementary Table 10 TRIPOD Checklist of prediction model development and validation

Section/Topic	Item	Checklist Item	Reported on Page number /Line number
Title and abstract			
Title	1	Identify the study as developing and/or validating a multivariable prediction model, the target population, and the outcome to be predicted	Page 1 / line 1-2
abstract	2	Provide a summary of objectives, study design, setting, participants, sample size, predictors, outcome, statistical analysis, results, and conclusions	Page 1-2 / line 19-41
Introduction			
Background and objectives	3a	Explain the medical context (including whether diagnostic or prognostic) and rationale for developing or validating the multivariable prediction model, including references to existing models	Page 2-3 / line 74-77
	3b	Specify the objectives, including whether the study describes the development or validation of the model or both	Page 3-4 / line 76-81
Methods			
Source of data	4a	Describe the study design or source of data (e.g., randomized trial, cohort, or registry data), separately for the development and validation data sets, if applicable	Page 3 / line 84-88<br>Page 4 / line 126-127
	4b	Specify the key study dates, including start of accrual; end of accrual; and, if applicable, end of follow-up	Page 3 / line 91-97
Participants	5a	Specify key elements of the study setting (e.g., primary care, secondary care, general population) including number and location of centres	Page 3 / line 85-86
	5b	Describe eligibility criteria for participants	Page 3 / line 86-89
	5c	Give details of treatments received, if relevant	Non relevant treatments
Outcome	6a	Clearly define the outcome that is predicted by the prediction model, including how and when assessed	Page 3 / line 101-102
	6b	Report any actions to blind assessment of the outcome to be predicted	Page 3 / line 104
Predictors	7a	Clearly define all predictors used in developing the multivariable prediction model,	Page 3-4 / line 104-124<br>Supplementary Table 1
	7b	including how and when they were measured Report any actions to blind assessment of predictors for the outcome and other predictors	Page 3-4 / line 104-124
Sample size	8	Explain how the study size was arrived at	Page 4 / line 153-154
Missing data	9	Describe how missing data were handled (e.g., complete-case analysis, single imputation, multiple imputation) with details of any imputation method	Page 4 / line 135-138
Statistical analysis methods	10a	Describe how predictors were handled in the analyses	Page 4 / line 139-149
	10b	Specify type of model, all model-building procedures (including any predictor selection), and method for internal validation	Page 4 / line 140-154
	10d	Specify all measures used to assess model performance and, if relevant, to	Page 4-5 / line 155-161
Risk groups	11	Provide details on how risk groups were created, if done	Page 4-5 / line 166-169
Results			
Participants	13a	Describe the flow of participants through the study, including the number of participants with and without the outcome and, if applicable, a summary of the follow-up time. A diagram may be helpful	Figure 1
	13b	Describe the characteristics of the participants (basic demographics, clinical features, available predictors), including the number of participants with missing data for predictors and outcome	Page 5 / line 181-189<br>Supplementary Table 1<br>Supplementary Table 2
Model development	14a	Specify the number of participants and outcome events in each analysis	Page 5 / line 181-185
	14b	If done, report the unadjusted association between each candidate predictor and outcome	Table 2
Model specification	15a	Present the full prediction model to allow predictions for individuals (i.e., all regression coefficients, and model intercept or baseline survival at a given time point)	Page 15 / line 517-549<br>Table 3<br>Supplementary Table 5<br>Figure 2
	15b	Explain how to use the prediction model	Page 8 / line 310-324
Model performance	16	Report performance measures (with CIs) for the prediction model	Page 6 / line 219-268<br>Table 3<br>Figure 2
Discussion			
Limitations	18	Discuss any limitations of the study (such as nonrepresentative sample, few events per predictor, missing data)	Page 8-9 / line 325-347

Interpretation	19b	Give an overall interpretation of the results, considering objectives, limitations, results from similar studies, and other relevant evidence	Page 7 / line 259-273
Implications	20	Discuss the potential clinical use of the model and implications for future research	Page 8 / line 310-324
Other information			
Supplementary information	21	Provide information about the availability of supplementary resources, such as study protocol, Web calculator, and data sets	Supplementary Excel algorithm
Funding	22	Give the source of funding and the role of the funders for the present study	Page 9 / line 363-365

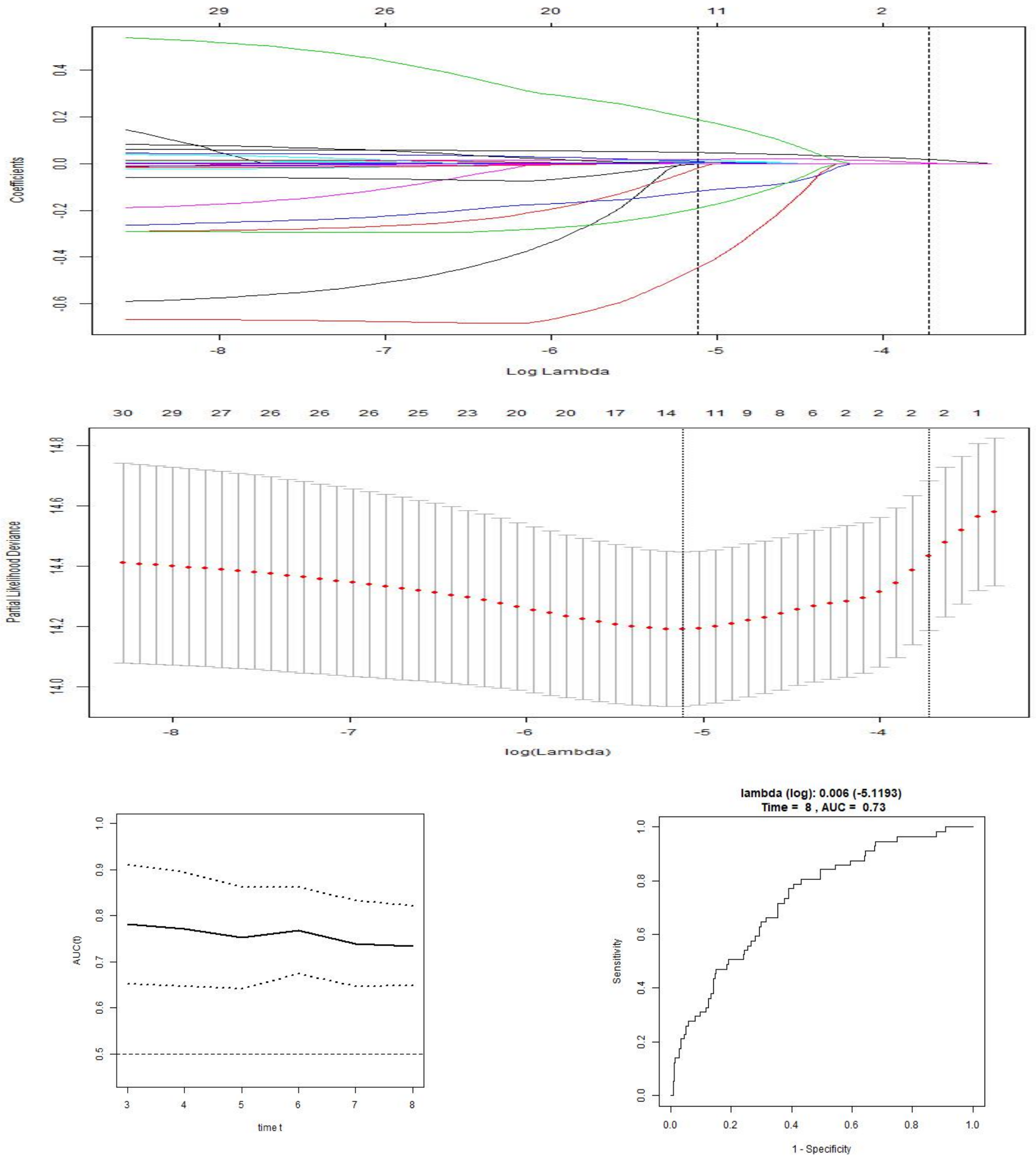

Supplementary Figure 1 LASSO regression analysis

Tuning parameter(lambda) selection in the LASSO model used 10-fold cross-validation.

Select lambda = lambda.min: 0.006 (-5.1193).

Formula for calculate score (not include Intercept): 0.04737*age(years)-0.01974*male+0.00817*BMI(kg/m^2)+0.01484*NC(cm)+0.01882*WC(cm)-0.1186*Without DM+0.00275*DM+0.00557*Hypertension with TOD-0.19092*Hypertension without TOD-0.44546*HDL-C(mmol/L)+0.18896*LDL-C(mmol/L)+0.00078*AHI(events/hour)

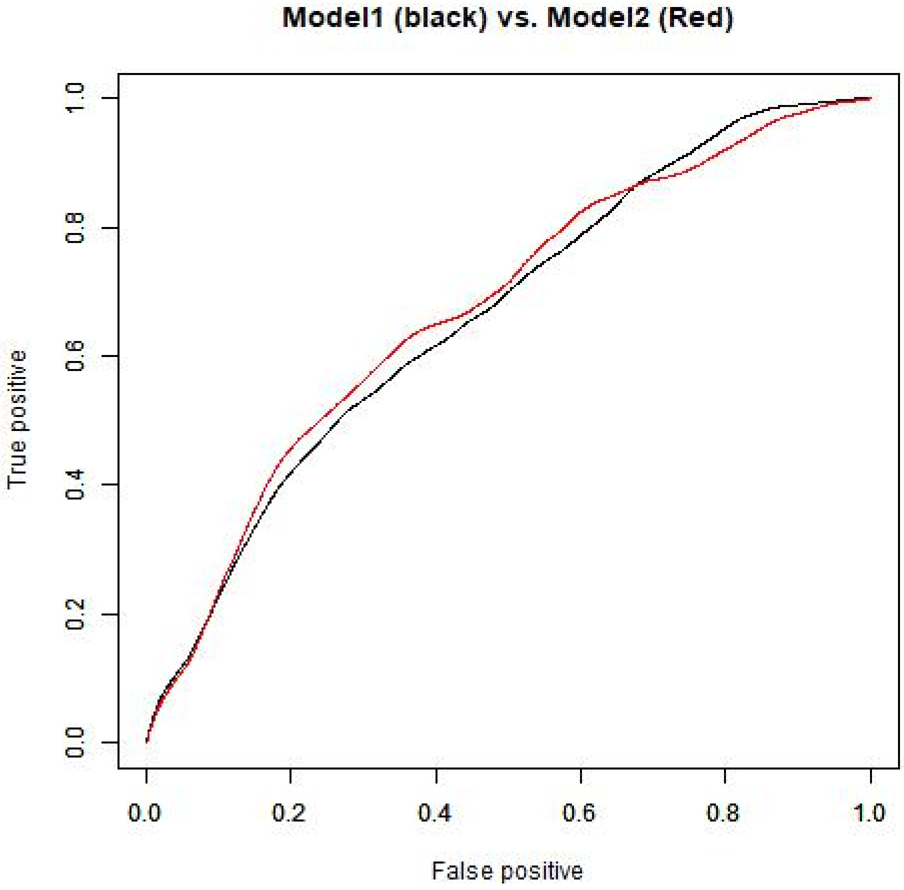

Supplementary Figure 2

Comparison of AUC between full model (model 1, AUC (95%CI):0.727 (0.670, 0.783)) and stepAIC selection model (model 2, AUC： 0.720 (0.662, 0.778))

Linear predictor (model1): 0.06259×Age(years) + 0.25873×gender(male) + 0.01602×NC(cm) + 0.01390×WC(cm) + 0.03086×BMI(kg/m^2)+0.34706×(hypertension with TOD) + 0.22671×(DM presence) − 0.79545×HDL-C(mmol/L) + 0.31234×LDL-C(mmol/L) + 0.00257×AHI(events/h).

Linear predictor (model2):0.05718×Age(years) + 0.03158×WC(cm) − 0.94899×HDL-C(mmol/L) + 0.29094×LDL-C(mmol/L) + 0.40965×(hypertension with TOD)

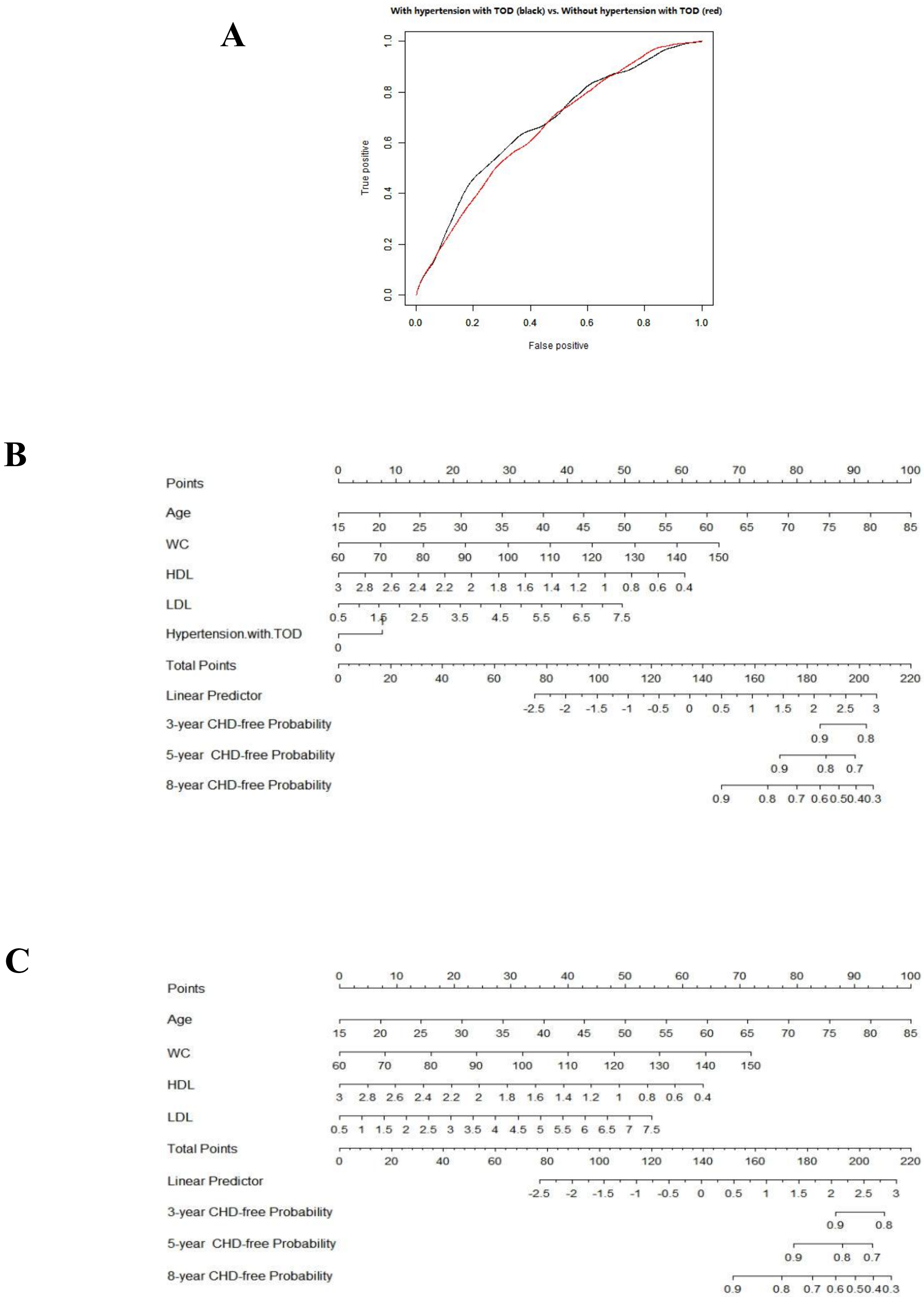

Supplementary Figure 3

A exploring analysis on whether with or without hypertension with target organ damage (TOD) in final nomogram model. (A) Comparison of AUC between with and without hypertension with TOD, (B) Nomogram model adding hypertension with TOD, (C) Nomogram model without this predictor.

A B

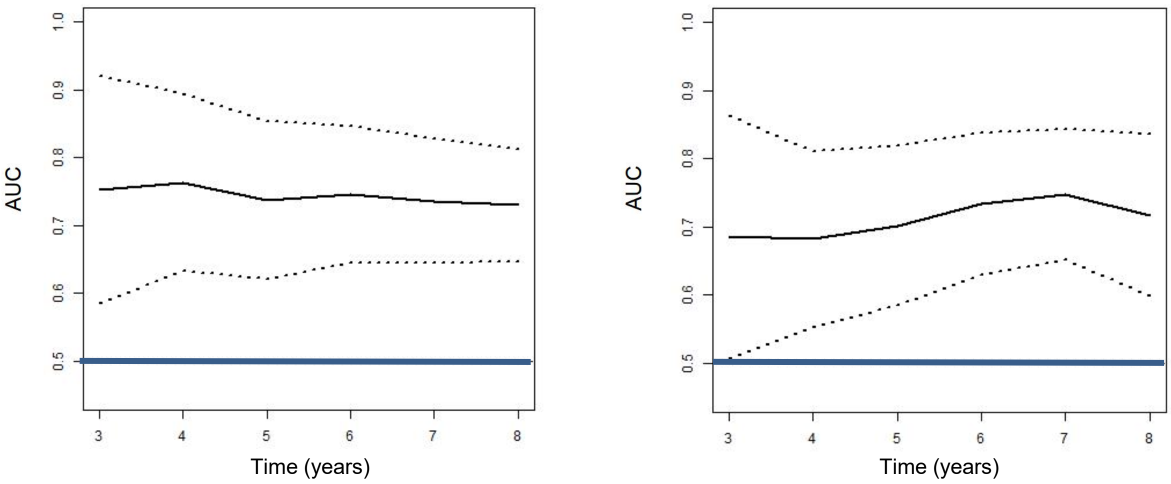

Supplementary Figure 4

Time-dependent area under the receiver operating characteristic (ROC) curve (AUC) to assess the nomogram's discrimination using 1000 bootstrap resampling internal validation in the training cohort (A) and in the validation cohort (B).

The solid lines depict the AUC at 3-, 4-, 5-, 6-, 7-, and 8-year of follow-up, and the dashed lines depict the AUC's 95% confidence interval, and the blue lines show the ROC curves are no better than chance.

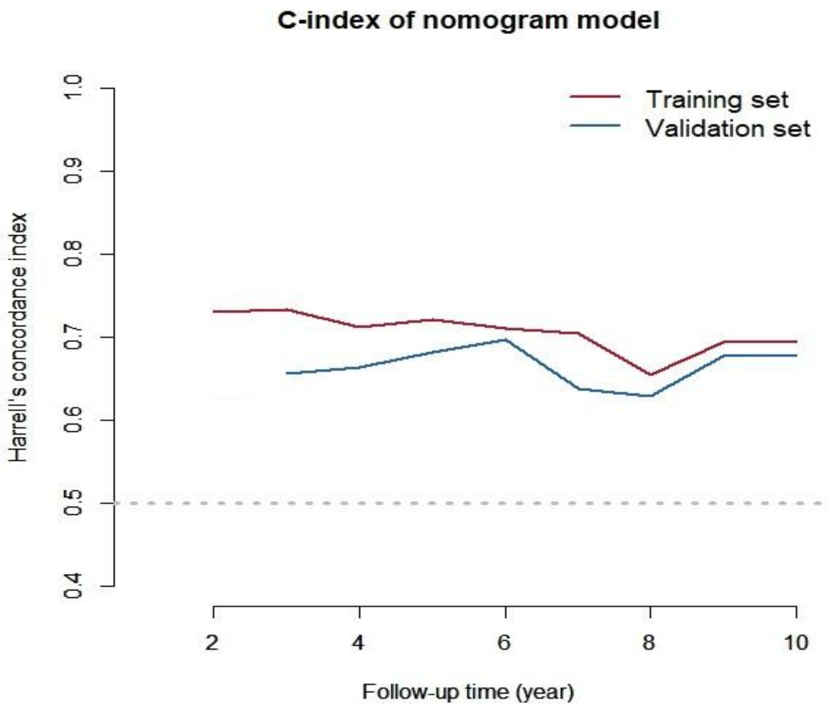

Supplementary Figure 5

The C-index at different follow-up years to assess the nomogram’s discrimination using 1000 bootstrap resampling internal validation in the training cohort and in the validation cohort
